# Interference-free Detection of Lipid-laden Atherosclerotic Plaques by 3D Co-registration of Frequency-Domain Differential Photoacoustic and Ultrasound Radar Imaging

**DOI:** 10.1038/s41598-019-48896-6

**Published:** 2019-08-27

**Authors:** Sung Soo Sean Choi, Bahman Lashkari, Andreas Mandelis, Jill J. Weyers, Aaron Boyes, Stuart F. Foster, Natasha Alves-Kotzev, Brian Courtney

**Affiliations:** 1 Center for Advanced Diffusion-Wave and Photoacoustic Technologies (CADIPT), University of Toronto, Toronto, ON M5S3G8 Canada; 2 Physical Sciences Department, Sunnybrook Research Institute, Toronto, ON M4N3M5 Canada; 3 Conavi Medical Inc., North York, ON M3B2V1 Canada

**Keywords:** Biophotonics, Photoacoustics

## Abstract

As lipid composition of atherosclerotic plaques is considered to be one of the primary indicators for plaque vulnerability, a diagnostic modality that can sensitively evaluate their necrotic core is highly desirable in atherosclerosis imaging. In this regard, intravascular photoacoustic (IVPA) imaging is an emerging plaque detection modality that provides lipid-specific chemical information of arterial walls. Within the near-infrared window, a 1210-nm optical source is usually chosen for IVPA applications because lipid exhibits a strong absorption peak at that wavelength. However, other arterial tissues also show some degree of absorption near 1210 nm and generate undesirable interfering PA signals. In this study, a novel wavelength-modulated Intravascular Differential Photoacoustic Radar (IV-DPAR) modality was introduced as an interference-free detection technique for a more accurate and reliable diagnosis of plaque progression. By using two low-power continuous-wave laser diodes in a differential manner, IV-DPAR could efficiently suppress undesirable absorptions and system noise, while dramatically improving system sensitivity and specificity to cholesterol, the primary ingredient of plaque necrotic core. When co-registered with intravascular ultrasound imaging, IV-DPAR could sensitively locate and characterize the lipid contents of plaques in human atherosclerotic arteries, regardless of their size and depth.

## Introduction

Atherosclerosis is a cardiovascular disease (CVD) in which lipid-rich plaques build up within the intima or the inner lining of an artery^1–3^. The complete cellular mechanism of the progression of atherosclerosis is not yet fully understood, but when plaque ruptures, interrupting the blood flow on site, it may lead to other more serious CVDs with high mortality rates such as myocardial infarction and stroke. While, in many cases, atherosclerosis can be asymptomatic for decades without causing any distinct clinical symptoms in patients^4,5^, plaques with higher risk of rupture are categorized as *vulnerable plaques* and many biochemical studies have confirmed a strong correlation between plaque vulnerability and their lipid composition^6–8^. Consequently, to detect the vulnerable plaques prior to possible life-threatening consequences of rupture, a diagnostic or imaging modality that can sensitively evaluate the lipid composition of plaques is highly desirable.

A few of today’s widely employed atherosclerosis imaging modalities include X-ray angiography, intravascular ultrasound (IVUS) and optical coherence tomography (OCT). These modalities assess atherosclerosis by evaluating the degree of stenosis, morphological changes, and superficial lipid composition of the plaques, respectively, but their reliability in sensitive detection of vulnerable plaques has long been questioned^4,9–12^. Angiography substantially lacks morphological or compositional information of plaques other than the extent of calcification. IVUS fails to deliver information that reliably differentiates between various forms of soft plaque^4,9,10^. OCT suffers from its limited penetration depth (~1 mm) due to severe light scattering through heterogeneous media and requires an additional step of flushing the vessel with an optically clear medium before performing image acquisition^4,11,12^.

Biomedical photoacoustics (PA) is an emerging optical-to-acoustic energy conversion technology where a subsurface target is optically excited with near-infrared light and imaged with optically-induced ultrasound signals^12–15^. By providing high optical contrast and superior ultrasonic depth-penetration at the same time, intravascular photoacoustic imaging (IVPA) is considered to be a promising modality that can provide the more reliable and detailed information needed for vulnerable plaque characterization^15–19^. In the Center for Advanced Diffusion-wave and Photoacoustic Technologies (CADIPT), University of Toronto, a PA imaging method has been under intense development based on frequency-modulated (chirped) optical excitation with low power continuous wave (CW) lasers and frequency-domain (FD) signal processing. This modality is called the Photoacoustic Radar (PAR) and has been shown to be competitive with conventional pulsed-based PA systems, providing high signal-to-noise ratio (SNR), sub-mm axial resolution and depth-resolved/molecularly specific optical contrast of the subsurface tissue chromophores, while utilizing low power irradiation and a narrowband detector^13,14^. Furthermore, use of CW optical sources in PAR allows flexible waveform engineering on the modulating optical signals that can lead to several unique or enhanced imaging features^20^. From this perspective, our earlier introduction of single-frequency wavelength-modulated differential photoacoustic spectroscopy^21–23^ led to the present multi-frequency intravascular differential PA radar (IV-DPAR) as an excellent example of the many waveform engineering possibilities for further advances in atherosclerosis imaging, beyond the physical limitations of the conventional PAR and pulse-based PA counterparts.

Conventional PA-based medical diagnostic technologies usually perform spectroscopic analysis or utilize single/multi wavelength laser(s) sequentially to obtain optical contrast from wavelength-specific absorption of subsurface targets^6–8,12^. This approach provides satisfactory results when target absorbers are the major source of PA signals in the region. However, its detection sensitivity and specificity become severely compromised when adjacent non-target chromophores have comparably high absorptions at that spectral region, generating undesirable PA signals that interfere with the target PA response in the acoustic domain. This is one of the main challenges that today’s IVPA applications (both pulse- and CW-based) face: while lipids show a clear absorption peak at 1210 nm, other common arterial tissues such as collagen and water exhibit some degree of absorption near 1210 nm as well^6^. The novelty of IV-DPAR presented here is based on the use of the second wavelength in real time with identical frequency modulation at a specific optical phase difference. Based on the widely available molecular absorption spectra of various biological materials found in human atherosclerotic arteries, lipids have a distinct absorption peak at ~1210 nm due to the second overtone of C-H bond vibration within the molecules while barely showing any at ~980 nm^6^. Other tissues found in arteries exhibit relatively similar absorptions at those two wavelengths because both wavelengths correspond to the vibrational overtone of O-H bonds of water molecules^6^. When two optical sources at those wavelengths are simultaneously modulated by an identical waveform but at an arbitrary optical wave phase difference (*φ*_*optical*_), the modulation signals, *r*_1210_(*t*) and *r*_980_(*t*), can be represented as:

1 $${r}_{1210}(t)=sgn[{\rm{s}}in(2\pi {f}_{1}t+\frac{\pi B{W}_{ch}}{{T}_{ch}}{t}^{2})],\,-\,\frac{{T}_{ch}}{2}\le t\le \frac{{T}_{ch}}{2}$$

2 $${r}_{980}(t)=sgn[{\rm{s}}in(2\pi {f}_{1}t+\frac{\pi B{W}_{ch}}{{T}_{ch}}{t}^{2}+{\phi }_{optical})],\,-\,\frac{{T}_{ch}}{2}\le t\le \frac{{T}_{ch}}{2}$$

where *sgn*(*x*) is the signum function, *f*_1_ is the starting frequency of the modulation chirp, *BW*_*ch*_ is the modulation chirp bandwidth and *T*_*ch*_ is the chirp duration. Two resulting PA output waves, *s*_1210_(*t*) and *s*_980_(*t*), would appear to be very similar with a certain acoustic phase difference (*φ*_acousic_) as:

3 $${s}_{1210}(t)={A}_{1210}[sgn[{\rm{s}}in(2\pi {f}_{1}t+\frac{\pi B{W}_{ch}}{{T}_{ch}}{t}^{2})]]+{\rm Z},-\,\frac{{T}_{ch}}{2}\le t\le \frac{{T}_{ch}}{2}$$

4 $${s}_{980}(t)={A}_{980}[sgn[{\rm{s}}in(2\pi {f}_{1}t+\frac{\pi B{W}_{ch}}{{T}_{ch}}{t}^{2}+{\phi }_{acoustic})]]+{\rm Z},\,-\,\frac{{T}_{ch}}{2}\le t\le \frac{{T}_{ch}}{2}$$

where *φ*_*shift*_ is the relative phase shift between two waves during the optical-to-acoustic energy conversion, *φ*_*acoustic*_ is the sum of *φ*_*optical*_ and *φ*_*shift*_, *A* is the magnitude of *s*(*t*) that is affected by absorption coefficient and optical fluence at each wavelength, and *Z* is possible system noise. *φ*_*shift*_ is mainly related to the optical properties of an absorber, but it also includes the effect of random phase shift caused by arbitrary system noise^15,21–23^. These two acoustic waves are related to each other by spatial and temporal constants, and undergo stationary interference in the acoustic domain. A general form of raw differential PA signals can be described as:

5 $${s}_{Diff}(t)={s}_{1210}(t)+{s}_{980}(t)$$

When *φ*_*optical*_ is carefully adjusted so that the system *φ*_*acoustic*_ becomes ~*π* after optical-to-acoustic energy conversion at each wavelength, *s*_1210_(*t*) and *s*_980_(*t*) will intrinsically undergo destructive interference and generate a single-channel differential PA signal, *s*_*Diff*_(*t*), upon generation^21–23^. During the calibration, when the amplitude ratio of *s*_1210_(*t*) and *s*_980_(*t*) from any noise source is adjusted to be ~1, the IV-DPAR system will highly suppress those unwanted absorptions or system noise to approximately zero baseline by means of complete destructive interference, while amplifying weak PA signals specifically emerging from the cholesterol contents of plaques. This study demonstrates this main principle of IV-DPAR by imaging pig and human atherosclerotic arteries *ex vivo* using the assembled IV-DPAR catheter prototype.

**Figure 1:**
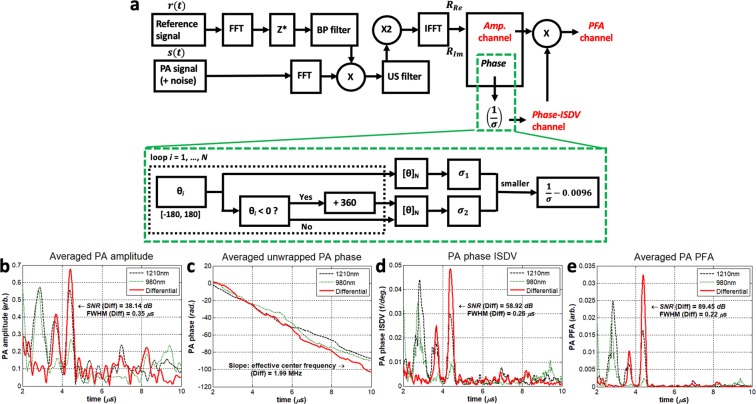
(**a**) Block diagram of the matched-filter pulse compression algorithm of IV-DPAR. The bandpass FD filter ensures that any frequency components outside the modulation bandwidth are eliminated during the signal-processing. Examples of IV-DPAR signal (from early-stage human plaque) in (**b**) amplitude, (**c**) unwrapped instantaneous phase, (**d**) phase-ISDV and (**e**) PFA channels. A total of 50 signals (N = 50) were obtained for this measurement point. By encoding statistical information of phase-ISDV on amplitude, the PFA channel showed dramatic improvement in SNR and FWHM axial resolution by approximately 135% and 37%, respectively, compared to the amplitude channel while utilizing the same raw data. FFT: Fast Fourier Transform, IFFT: Inverse Fast Fourier Transform, Z*: complex conjugate, Re: Real component of *R*, Im: Imaginary component of *R*, X: multiplication, σ: standard deviation, BP filter: Bandpass filter, Amp.: Amplitude, Phase-ISDV: Phase Inverse Standard Deviation, PFA: Phase-filtered Amplitude.

The block diagram of an IV-DPAR signal processing algorithm is illustrated in Fig. 1a, where an analytic signal is generated after the matched-filter and pulse compression processing of the received PA information in FD. Assuming there exists additive stochastic noise in the system, this algorithm maximizes the system SNR^13,14^. The amplitude channel directly provides magnitude and delay time (or depth using the speed of sound in a medium) information of the light-tissue interaction. However, the unwrapped correlation phase is linear over time with a slope given by the effective center frequency of the modulating chirp and provides no practical information for endoscopic applications. Instead, appreciating the fact that the instantaneous correlation phase value is fixed when the imaging configuration, like a detector-target distance, is unchanged, its inverse standard deviation from N consecutive measurements can be evaluated to extract meaningful statistical information about the presence of the target. In other words, when there is an actual signal, even with a very small magnitude, multiple phase signals have relatively low standard deviation and therefore, its inverse of the mean phase value locks at the corresponding value. On the other hand, without the presence of an actual signal, phase signals are dominated by random system noise. Their standard deviation is high, and consequently, its inverse is highly suppressed to the baseline. In addition, such fluctuating phase from background fall in the uniform distribution between -π and π. When N → ∞, the reciprocal of its σ is approximated as 0.0096 (1/degree) and, in order to reduce the baseline of this channel, the entire Phase-ISDV plot can be arithmetically subtracted by this constant. Such processing simply improves signal SNR while keeping the information intact. This phase inverse-standard deviation (Phase-ISDV) channel evaluates and further gate the pure correlation phase channel at a much higher sampling frequency of the system. Therefore, the Phase-ISDV channel exhibits relatively higher SNR and axial resolution compared to the corresponding amplitude channel. Encoding the statistical information of Phase-ISDV on the amplitude channel, the phase-filtered amplitude (PFA) channel further enhances SNR and axial resolution of the target PA signals. An example of human plaque signals from various IV-DPAR channels is shown in Fig. 1b–e. While details of the employed experimental set-up will be described in the following section, the PFA channel showed approximately 135% and 37% improvement in its SNR and full width at half maximum (FWHM) axial resolution, respectively, compared to the corresponding amplitude channel. With the chirp modulation frequency linearly swept in 1–5 MHz, the slope of the unwrapped phase revealed the effective center frequency of the generated PA signal to be ~2 MHz, but this channel provided no other useful information needed for plaque detection.

## Results

### Intravascular differential photoacoustic radar (IV-DPAR) signal analysis from the artificial plaque phantom

**Figure 2:**
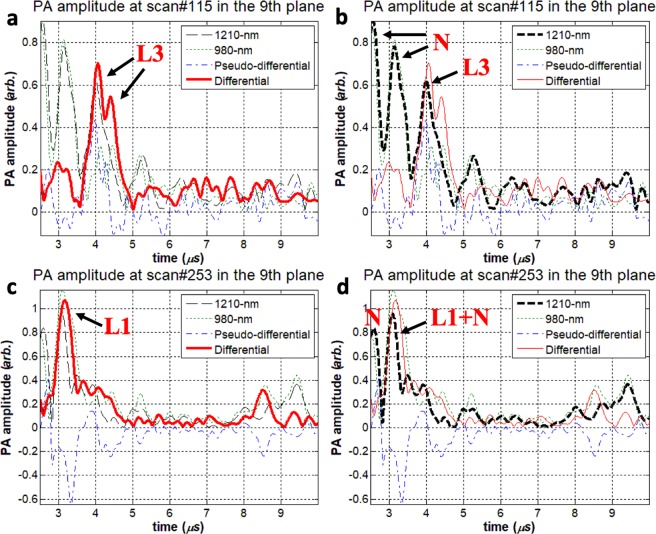
Artificial plaque lump 3 (L3) signal was located very close to strong RF noise at scan #115 in the 9^th^ imaging plane. From this measurement, (**a**) IV-DPAR amplitude and (**b**) LF single-ended 1210-nm PAR amplitude was highlighted. At scan #253 in the 9^th^ imaging plane, the L1 signal was completely superposed with the local RF noise. From this measurement, (**c**) IV-DPAR amplitude and (**d**) LF single-ended 1210-nm PAR amplitude was highlighted. In both situations, IV-DPAR could successfully extract cholesterol signals from the background while the single-ended 1210-nm PAR mode was severely affected by the adjacent noise. N: RF + arterial wall noise.

Specific low-frequency (LF) experimental data sets from the pig artery phantom and the 4 MHz transducer were selected to investigate how IV-DPAR would behave differently from its single-ended and pseudo-differential PAR counterparts when cholesterol molecules are located very close to noise signals, thereby undergoing signal interference. The pseudo-differential approach is one of the popular algorithms that is widely employed by multi-spectral pulse-based IVPA systems when trying to obtain their version of differential PA signals^6,12,24^. This algorithm does not require simultaneous wavelength-modulation of multiple optical sources, but instead, applies simple subtraction of the resulting PA signals to filter out the desired cholesterol signals from noise. One measurement point from the circumferential scan (#115 in the 9^th^ azimuthal plane) served as an example of such a case where one of the four artificial plaques injected, Lump 3 or L3, was located in the immediate neighborhood of strong RF noise. It should be noted that the amplitude channels were used for this comparison as they provided the signals with the least degree of post-processing. As shown in Fig. 2a, IV-DPAR was only sensitive to lipid and could recover both the front and back surfaces of L3 accurately. However, the same cholesterol peaks could not be easily extracted from the corresponding single-ended 1210-nm mode in Fig. 2b due to the adjacent noise. In addition, the second cholesterol peak that corresponds to the back surface of the volume was lost possibly due to the interfering noise. The pseudo-differential channel generated some non-physical negative outputs as it utilized a simple subtraction algorithm between the two independent PAR signals. A similar result was also observed when the L1 signal was completely superposed with the local RF noise in Fig. 2c,d.

While such undesirable signal interferences are very likely to occur during sub-mm scale IV plaque imaging, to the best of our knowledge they have never been thoroughly investigated with conventional time-domain (TD) pulse-based IVPA systems. In many pulse-based studies, only the single wavelength that coincides with the absorption peaks of lipids (1210 nm or 1700 nm) has been utilized to characterize the cholesterol contents of plaques^16,18,25^. As the bipolar shape of pulsed IVPA signals reduces the probability of differentiating between two depth-wise adjacent chromophores^14^, the detection accuracy and reliability of such single-wavelength modalities are severely compromised when lipids are located near non-target absorbers. Various forms of spectroscopic analysis have been tried with pulse-based IVPA systems to differentiate plaques from surrounding tissues^7,17^, but they require time-consuming multi-wavelength measurements for every data acquisition point and each wavelength is still evaluated independently, ignoring the possible effects of signal interferences. Many pulse-based IVPA systems employ the multi-spectral approach and evaluate the arithmetic difference between the resulting individual PA signals to extract cholesterol information^6,12,24^. As pulse-based IVPA systems do not involve FD signal processing which generates real/imaginary components of the signal and sidelobes as a processing artifact, the interference between two adjacent PA signals is simply linear in this case. Therefore, this modality is expected to generate similar interference-free lipid signals as IV-DPAR. However, such approach in pulsed IVPA is not as reliable as the wavelength-modulated differential method of IV-DPAR for many reasons. Pulse-based IVPA systems require wideband detection, so multi-channel processing amplifies wideband noise along with other types of intrinsic system induced noise (i.e. electric noise of the transducer). Furthermore, when successive trains of ns-long pulses are applied with large temporal gaps between individual pulses, there can be no synchronous simultaneous signal processing to simply subtract interference in multi-spectral PA imaging. Under time sequence conditions, any undesirable time-dependent effects interfere with actual absorber-related signals, especially in *in vivo* imaging where the imaging environment changes continuously such as with breathing motion, and thus reliable real-time imaging becomes challenging. An optimal method to obtain true cholesterol information during endoscopic imaging is to dispose of possible interfering signals before actual interferences are sensed by the detector. This can be easily achieved using CW-based IV-DPAR through simultaneous wavelength-modulated differential suppression.

### Artificial plaque signal analysis

**Figure 3:**
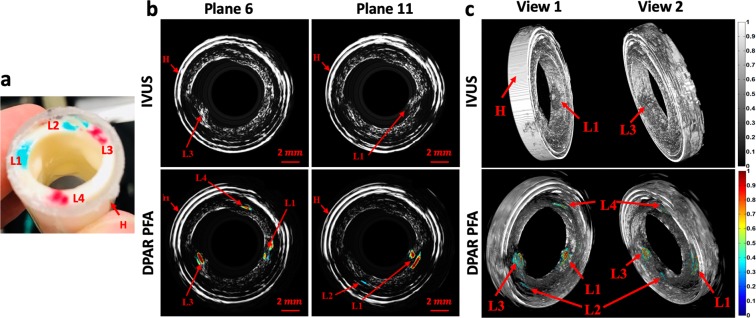
(**a**) Picture of the pig artery phantom with four artificial plaques. (**b**) Cross-sectional IVUS and co-registered LF IV-DPAR/IVUS PFA images in the 6^th^ and 11^th^ azimuthal planes. (**c**) 3D IVUS and co-registered LF-IVDPAR/IVUS images of atherosclerotic artery phantom. The contrast of background IVUS images was arbitrarily adjusted to highlight overlapping PAR signal traces. H: generic plastic holder.

Figure 3b (top) shows selected cross-sectional IVUS images of the pig artery phantom with four artificial plaques or lipid lumps (L). As shown in Fig. 3a, L1 and L3 were already large enough to be visible with the naked eye and could also be easily detected by their morphology in the purely IVUS mode. However, the IVUS images alone could not reveal the detailed depth distribution of the lipid within artificial plaques. In addition, L2 and L4 were not visible in the IVUS mode due to their small size and relatively large injection depth as US signals lack specificity to cholesterol. On the other hand, when the PFA information of the LF IV-DPAR mode was co-registered with the corresponding IVUS images, the exact locations and depth distributions of all four artificial plaques became visible as shown in Fig. 3b (bottom), regardless of the injection amount and depth, thus indicating excellent sensitivity and specificity of the IV-DPAR modality. The foregoing procedure was further extended to 3D volume imaging. The 3D IVUS image in Fig. 3c (top) could locate L1 and L3 based on their protruding morphology but could not identify other lumps at any angle of view because they were morphologically indistinguishable from the wall. However, all four cholesterol sites could be successfully detected from the combined 3D LF IV-DPAR/IVUS image in Fig. 3c (bottom) as the IV-DPAR signals were spectroscopically only sensitive and specific to cholesterol molecules.

### Human atherosclerotic artery imaging

**Figure 4:**
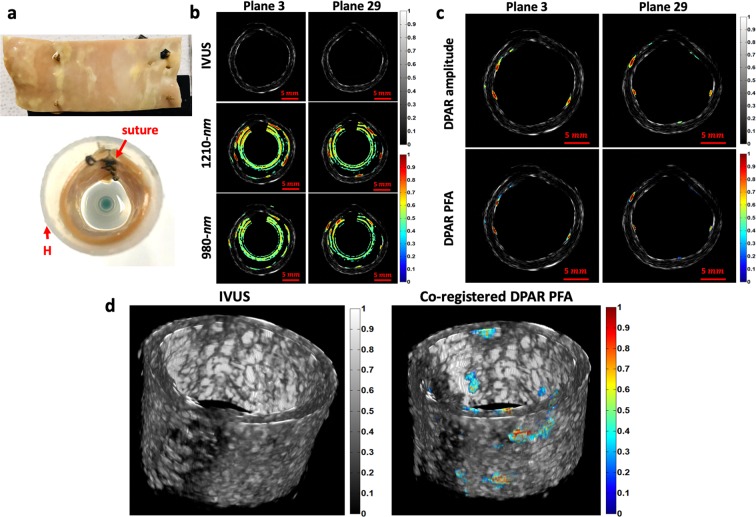
(**a**) Picture of sutured early-stage human atherosclerotic artery. (**b**) Cross-sectional IVUS and co-registered LF single-ended PAR/IVUS amplitude images of the early-stage human atherosclerotic artery at two different azimuthal planes. (**c**) Co-registered LF IV-DPAR/IVUS amplitude and PFA images of the same sample planes. While cholesterol-specific information was available with no ambiguity, the PFA channel provided greater dynamic range. (**d**) Purely 3D IVUS and co-registered 3D LF IV-DPAR/IVUS PFA views of the early-stage human atherosclerotic artery. H: generic plastic holder.

Among 40 azimuthal planes imaged, planes 3 and 29 were selected to present the cross-sectional images of the early-stage human atherosclerotic artery shown in Fig. 4a. These two were very suitable examples as they contained a few well-differentiated plaques along the plane. Figure 4b shows the results from the conventional imaging modalities first. While IVUS could provide detailed structural information of the artery, it was rather infeasible to identify the plaques from these images. Furthermore, the early-stage atherosclerotic plaques did not raise the intimal surface much, making the morphology-based differentiation even more difficult in the IVUS mode. The LF single-ended PAR modes could add one extra layer of chemical-specific information on the corresponding IVUS images. However, due to the presence of strong RF and arterial wall noise, it was rather challenging to evaluate the true forms of plaques. Figure 4c shows the corresponding LF IV-DPAR results. As the undesirable noise was suppressed, the true forms of cholesterol information could be revealed with excellent sensitivity and specificity. Without any noise signals in proximity, these signals were free from undesirable signal interference that could potentially distort the conveyed information. As expected, the PFA channel exhibited superior dynamic range to the amplitude channel, improving the image contrast. A series of cross-sectional images were then reconstructed into a 3D volume image as shown in Fig. 4d. These images confirmed how IV-DPAR enabled an accurate detection of plaques that requires no further analysis and interpretation. Since all the processing was done intrinsically, the cholesterol traces were the only color-coded shapes on the image. On the other hand, since those early-stage plaque regions were not anatomically apparent, no accurate evaluation could be made in the gray-scaled IVUS images.

**Figure 5:**
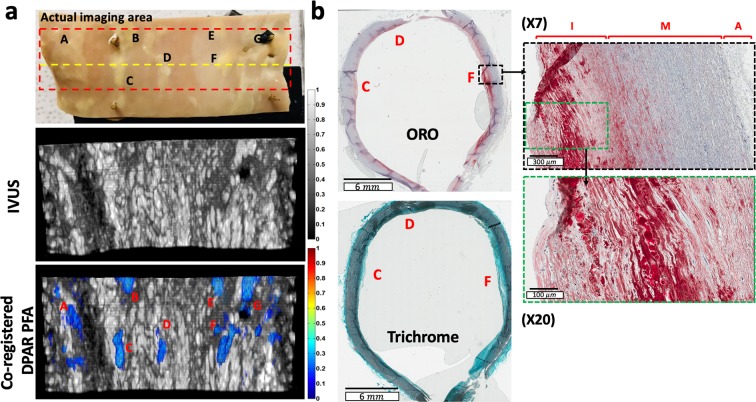
(**a**) Picture of the early-stage human atherosclerotic artery and the corresponding 3D flattened images of IVUS and co-registered LF IV-DPAR/IVUS. The flattened images were arbitrarily opened up from the reconstructed circular image in Fig. 4d. (**b**) ORO and trichrome staining results of the thin layer of the early-stage human atherosclerotic artery (indicated by the yellow dashed line on picture in (**a**). ORO: Oil Red O.

While the resulting LF IV-DPAR images were shown to be quite promising in selective plaque detection, it was still necessary to confirm if what appeared to be plaques in the IV-DPAR images were truly the plaques. For this purpose, two different validation approaches were taken. The first approach was visual validation. The early-stage plaques could be visually differentiated to some extent by their texture and color. They appeared as irregular yellow nodules caused by deposition of lipids. As shown in Fig. 5a, the LF IV-DPAR results matched very well with the actual shapes and positions of different lipid nodules. Since all the nodules were formed very close to the intimal surface in this early-stage sample, such comparison was straightforward. The second approach was histological validation. A thin layer used for this validation included three well-distinguished early-stage plaques as indicated by the yellow dashed line in Fig. 5a. The tissue was sliced from the circularly-shaped artery, and therefore the resulting histology results were also circular in shape. The ORO staining result in Fig. 5b confirmed that the yellowish nodules seen from the sample were truly composed of lipid. The trichrome staining result showed the general distribution of collagen in this sample.

**Figure 6:**
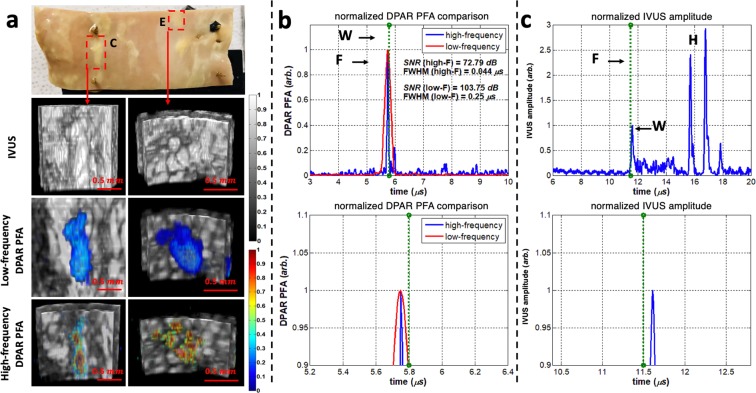
Comparison between the LF and HF IV-DPAR (**a**) images of plaques C and E in the early-stage human atherosclerotic artery, (**b**) signal traces of plaque C in the PFA channel and (**c**) in the IVUS channel. W: arterial wall, F: front surface of necrotic core, H: generic plastic holder.

Two plaques, C and E, were randomly chosen to demonstrate additional details that the high-frequency (HF) IV-DPAR system can provide. As shown in Fig. 6a, the LF IV-DPAR images indicated the locations and shapes of the irregular plaques with excellent contrast and accuracy. However, due to limited axial resolution, they appeared as bulged homogeneous lumps. When the same plaques were scanned with HF IV-DPAR in Fig. 6a, on the other hand, the micro-distributions of cholesterol molecules were revealed. Such details may not be needed when trying to simply locate plaques within an artery but may help predicting future behavior of plaques. If desired, these images may be easily superimposed in a post-processing manner to provide a more complete view of imaging plaques^26^. More detailed comparison between the LF and the HF IV-DPAR systems could be made in terms of their signal traces in Fig. 6b,c. For this purpose, one of plaque C measurements was selected. The locations of the arterial wall and the cholesterol core could be evaluated by IVUS and IV-DPAR, respectively, with regards to their front surfaces. The results showed that the thin layer of cholesterol was formed on the intimal surface. While the SNR of the PFA channel was superior to that of the amplitude channel in each system, the LF system exhibited higher SNR in general. It should be noted that in the HF IV-DPAR PFA channel, the axial resolution of 0.044 μs or 65 μm (assuming the speed of sound in water to be ~1.48 mm/μs^14^) was achieved.

In this study, IV-DPAR was developed as a reliable CW-based PA imaging method that can accurately detect true cholesterol signals without being affected by undesirable local background absorption interferences. Simultaneous two-wavelength modulation resulted in destructive interference between the two resulting PA waves, leading to effective intrinsic suppression of undesirable signals and noise background. Unlike the PAR and the single-ended pulse-based IVPA techniques, this modality was proved to be highly sensitive and selective to spectroscopically defined cholesterol targets. This modality was unlike conventional pulse-based multi-spectral PA modalities that rely on simple subtraction algorithms of sequentially processed signals. When co-registered with IVUS in 3D, IV-DPAR could successfully reveal accurate interference-free locations and radial depth-profiles of plaque necrotic cores in the tested *ex vivo* samples with excellent sensitivity and specificity, even when the regions were not anatomically apparent.

The LF system was shown to be ideal for fast acquisition of the overall plaque information. It required fewer signals for averaging and was less sensitive to the surface morphology of the imaging targets. However, it exhibited some limitations in realizing small details of the plaques due to limited axial resolution even at the PFA channel. While SNR was compromised, the HF system could resolve those missing details with its much higher axial resolution.

The focus of this study was to develop and demonstrate the unique imaging potential of IV-DPAR where imaging was performed with a bench-top catheter prototype in deionized water. It should be, however, acknowledged that the clinical catheterization technology is already well developed in other imaging fields such as IVUS and OCT, and many interests have already been raised in making pulse-based IVPA techniques real-time by optimizing hardware/software components of the system^16,25^. In transition to the clinical *in vivo* imaging system, IV-DPAR is expected to be easily integrated to such clinically-accepted catheter systems with minimum additional investment since the novelty of IV-DPAR resides in the use of simultaneous optical modulation and the corresponding FD signal processing technique. In *in vivo* intravascular imaging scenario in presence of blood, the SNR of the response signal is expected to be lower due to increased light attenuation by hemoglobin. However, as demonstrated by other pulse-based IVPA groups, PA still generate promising chemical-specific images in blood, especially in intravascular environment where the photon travel path is limited to a few millimeters at most^6,27^. Furthermore, in IV-DPAR, the effect of blood attenuation is expected to be minimized due to the arbitrarily suppressed system baseline that is to be only sensitive to the presence of lipids. While much more preclinical/clinical research remains to follow, the unique imaging capability of IV-DPAR holds strong diagnostic potential for atherosclerosis.

Further improvement of this technique is readily achievable by allowing more optical power in the system. Due to the technical limitation of the current optical modulators, the optical fluence of current IV-DPAR is about half of the maximum permissible exposure (MPE) level allowed at human tissue^28^. With appropriate advancement in optical hardware, when more optical power is allowed under the safety limit at ~MHz modulation frequencies, the SNR of IV-DPAR is expected to improve further even with shorter irradiation or a smaller number of signal averaging.

## Methods

**Figure 7:**
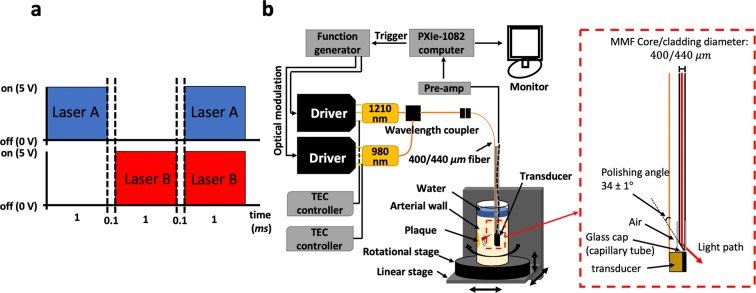
(**a**) Each channel of the dual-channel arbitrary waveform generator was coded in a [1, 0, 1] and [0, 1, 1] manner, where 0 and 1 represent the laser off and on, respectively. While the duration of each chirp was 1 ms, 0.1 ms gaps were added in between to ensure the single-ended and differential PA signals are well-separated. (**b**) Block diagram of the general experimental set-up and the schematic of the IV-DPAR catheter tip.

Two CW optical sources, Laser A (λ_A_ = 1210 nm; RPMC, MO, USA) and Laser B (λ_B_ = 980 nm; RPMC, MO, USA), were employed to excite an imaging target. Both diodes were integrated with customized fast-modulation drivers (Fast Analog Carlsbad, CA, USA) that could modulate the lasers with reliable square waveforms up to approximately 24 MHz. Among various optical waveforms (i.e. sinusoidal and pulse trains), the square waveform was chosen for this study as it was shown to provide extra gain in the output SNR due to the difference in the frequency spectrum of the excitation signal involved in PA generation^20^. A dual-channel arbitrary waveform generator (33522B; Agilent, CA, USA) was used to modulate the two drivers simultaneously, as shown in Fig. 7a, so that all possible single-ended and differential PA signals could be obtained sequentially from the identical location of the sample. Each chirp was linearly modulated by a square waveform in the 1–5 MHz range for LF imaging or 3–14 MHz for HF imaging at an optical wave phase difference of 180° with less than ±0.01° deviation. While the optical power of the 1210-nm laser was set to be maximum at ~220 mW, that of the 980-nm laser was arbitrarily tuned (by controlling the input current of the driver) to achieve *R* = 1 for undesirable PA signals from noise. The waveform generator was synchronized with a National Instruments (NI) data acquisition card (NI PXIe-5122, TX, USA) by an external trigger every 4 ms. Two TEC controllers (Arroyo 5305; Arroyo Instruments, CA, USA) were employed to stabilize the diode temperature at 21.5 °C (room temperature) during the operation with less than ±0.01 °C deviation. The two fibers from each diode were coupled into a single fiber with a customized wavelength coupler (WDM-12P-111-980/1210-400/440-QMQMQM-35-555-3A-1, OZ Optics, Ottawa, ON, Canada). The single output fiber was then connected to a 400/440 μm (core/cladding) bare optical fiber with a refractive index of ~1.44 and ~34° cut at the tip (OZ Optics, Ottawa, ON, Canada) to deliver the light to the target location within the vessel by total internal reflection. During clinical intravascular imaging, the catheter would need to be submerged in rhythmically flowing blood within the vessel. Since blood has a relatively high refractive index of ≥1.41 at the wavelengths of our interest^29^, the polished end of the optical fiber was covered with a glass cap to provide an air medium inside with the lower refractive index than the fiber core, ensuring efficient total internal reflection at the fiber tip with minimum energy loss. For PA signal acquisition, two 1.5 mm × 1.5 mm customized ultrasonic transducers with 4-MHz (LF) and 14-MHz (HF) center frequencies were utilized (Sunnybrook Research Institute, Toronto, ON, Canada). For IVUS co-registration, the HF transducer was employed both as an emitter and a receiver while an emitted acoustic signal was modulated with 1-ms-long 3–24 MHz sinusoidal chirp. The obtained IVUS signal was then processed by the same FD algorithm shown in Fig. 1a as an ultrasonic radar. As shown in Fig. 7b, the transducer and the 400/440 μm optical fiber were aligned together and assembled into the catheter prototype with diameter ~4 mm. The received signal was amplified by 40 dB (5676, Olympus Panametrics, CA, USA). For the LF system, 50 signal records were obtained for each point during the measurement to evaluate the average and ISDV. For the HF system, 200 signals were obtained to compensate the compromised SNR. The general experimental set-up is depicted in Fig. 7b.

An artificial plaque phantom was prepared using a porcine artery (descending aorta resected from healthy Yorkshire pigs) with inner diameter of ~9.7 mm (Sunnybrook Research Institute, Toronto, ON, Canada). An arbitrary amount of cholesteryl-oleate powder (abcr GmbH, KA, Germany) was melted in a ~50 °C water bath, and then micro-injected within the porcine vessel wall at four different locations using a prewarmed syringes. Injection locations were marked on the cross-sectional edge of the vessel using tissue paint (Polysciences Inc., PA, USA). The amount and depth of injections varied, simulating plaque necrotic cores of different sizes at different depths. As shown in Fig. 3a, L1 and L3 were visually differentiable from the arterial wall, but L2 and L4 were not due to their small size. One piece of early-stage human atherosclerotic artery (aorta) was obtained for human tissue imaging (Sunnybrook Research Institute, Toronto, ON, Canada). Aorta tissue was obtained post-autopsy and has been sliced open longitudinally as part of the post-mortem exam. The tissue had been stripped of patient identifiers and no patient information was collected. Since the artery was sliced open, it was re-circularized and sutured together using a 2–0 silk suture (Covidien plc, LET, Ireland) to recreate the circular shape that simulate more realistic geometry of an intact artery as shown in Fig. 4a. In their circular shape, the lumen diameters were ~13 mm for both. The thickness of the early-stage human atherosclerotic artery was measured to be ~2 mm. The sutured sample was placed in the generic plastic holder to be filled with deionized water for acoustic coupling. All animal and human tissues used for this study were obtained and the associated experimental protocols were followed under the approval of the Sunnybrook Research Ethics Board and the University Health Network Research Ethics Board.

All samples were placed at the center of the rotational stage. While the catheter was fixed near the center of rotation, the loaded sample was rotated with 1.00 ± 0.04° resolution using a motorized rotation stage (PRMTZ8/M and KDC101, Thorlabs, NJ, USA) to obtain a cross-sectional image of the inner surface including radial depth profiles. Multiple azimuthal planes were imaged with 500 μm resolution using a motorized linear stage (Z825B and TDC001, Thorlabs, NJ, USA). The differential tuning was performed to suppress the system RF and undesired PA noise from the arterial walls in general. Each set of images was normalized by the pixel with the maximum magnitude within each dataset so that the resulting 3D volume images are presented in the dynamic range between 0 and 1. During the signal processing of the human artery dataset, the PA signals from the plastic holder and the suture were arbitrarily filtered. Data acquisition, signal processing, motion control and image reconstruction were controlled by a single LabVIEW 2014 program developed in the CADIPT. Histology validation was performed on the human tissues at the Pathology Research Programs laboratory, University Health Network, Toronto. The plaque-containing sections of tissues were sliced to 9 μm and 7 μm for staining with Oil red O (ORO) and trichrome, respectively. ORO was used to stain cholesterol molecules specifically while trichrome was used to show overall distribution of collagen in the same region. The stained tissues were then digitized at the Digital Pathology Laboratory, University Health Network, Toronto.

## Data Availability

The datasets generated during and/or analysed during the current study are available from SS.S.C. on reasonable request.
